# Urinothorax: A rare complication of vertebral body tethering

**DOI:** 10.1016/j.radcr.2025.02.021

**Published:** 2025-03-08

**Authors:** Alexa Pisciotti, Daniel Hsu, Muhammad Omar Afridi, Sharon Underberg-Davis

**Affiliations:** a Rutgers Robert Wood Johnson Medical School, Department of Radiology, New Brunswick, NJ, USA; b Beth Israel Deaconess Medical Center, Department of Radiology, Boston, MA, USA; c Harvard Medical School, Department of Radiology, Boston, MA, USA

**Keywords:** Anterior vertebral body tethering (AVBT), Nephropleural fistula, Urinothorax, Case report

## Abstract

Anterior vertebral body tethering (AVBT) offers an alternative to traditional spinal fusion for adolescent idiopathic scoliosis (AIS) that preserves spinal mobility. However, its novelty means that there is limited data on its associated complications. This case report details a rare postoperative complication: nephropleural fistula resulting in urinothorax occurring in a patient with AIS undergoing AVBT. This patient required extended hospital stays and multidisciplinary management. This occurrence underscores the need for identifying and managing unusual complications related to AVBT. Enhancing awareness can improve patient safety and optimize procedural outcomes in the evolving landscape of AIS treatment.

## Introduction

Anterior vertebral body tethering (AVBT) has ignited a paradigm shift in the treatment of adolescent idiopathic scoliosis (AIS) in skeletally immature patients providing an alternative to spinal fusion for those patients with incomplete natural growth. Using a similar technique to a spinal fusion procedure, an internal brace is created that corrects the spine without fusion, guiding the growing spine into alignment and allowing for increased spinal mobility and flexibility post-operatively [1].

AVBT was first described with the treatment of a patient who was diagnosed with juvenile idiopathic scoliosis at age five [2], with significant correction of thoracic curvature. The technique was later approved by the US Food and Drug Administration in July 2019 and has since been increasingly used for treatment of AIS. Because AVBT is a relatively new technique, data is limited, and complications have not been well described. To the best of our knowledge, occurrences of nephropleural fistula following AVBT have not been well documented. This case report describes a patient with AIS treated with AVBT which was complicated by postoperative formation of nephropleural fistula and urinothorax.

## Case

**Fig. 1:**
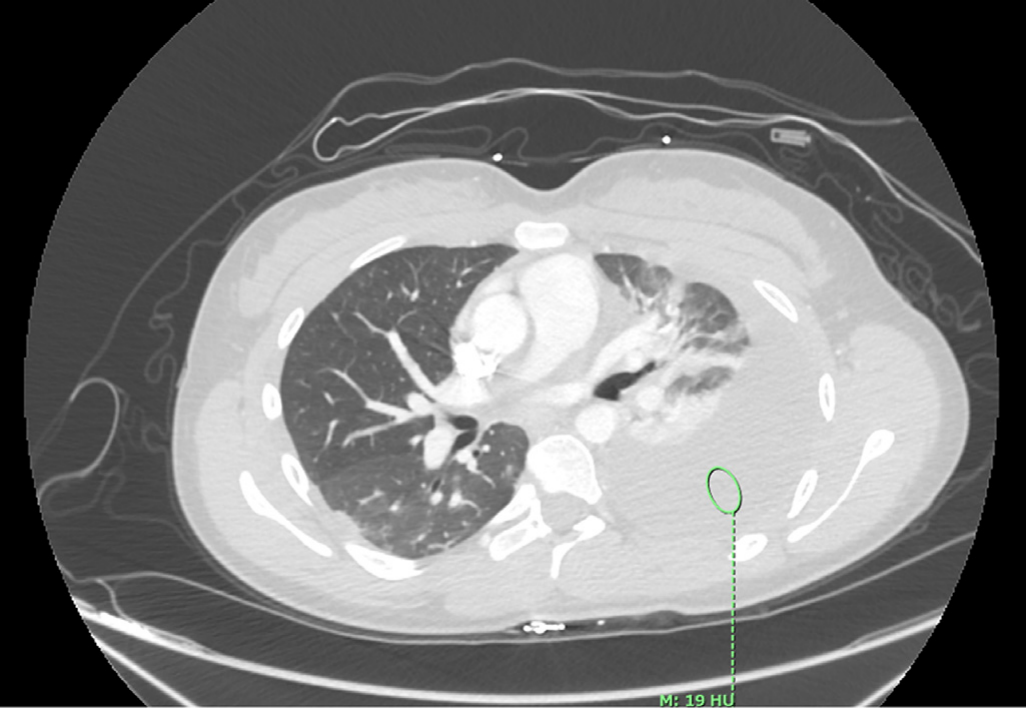
CT chest shows low density large pleural effusion (circle).

Sixteen-year-old female with a past medical history of idiopathic scoliosis status post anterior scoliosis correction surgery 4 years prior and exercise-induced asthma presented as an outpatient for progressive thoracolumbar scoliosis. The decision was made to proceed with a revision, mini-open T10-11, thoracotomy exposure T11-L4 with mini subfascial approach L3-4, a new revision left anterior spinal fusion approach T11-L4 partially reduced 5% with T11/12- L3/4 mobilizing anterior annular and ligament releases partial diskectomies, as well as new separate and additional left anterior spinal instrumentation T11-L4. The patient tolerated the procedure well and was discharged from the hospital on postoperative day (POD) four. Two days following discharge (POD 6), she presented to the emergency department at an outside hospital with acute shortness of breath and left sided chest pain. She was transferred to our hospital for further management. A left-sided chest tube was placed and pleural fluid analysis was compatible with a hemothorax. On POD 7, a CT scan of the chest without contrast revealed a worsening complex fluid collection in the left pleural space measuring 20 Hounsfield units (Fig. 1), with overlying pneumothorax and complete compressive atelectasis of the left lower lobe and near complete atelectasis of the left upper lobe. By POD 13, the patient continued to have unusually high output from the left sided chest tube, which was now draining serosanguinous fluid. At this time, the pleural fluid was sent for further analysis and showed a creatinine level of 11, compatible with urine.

**Fig. 2:**
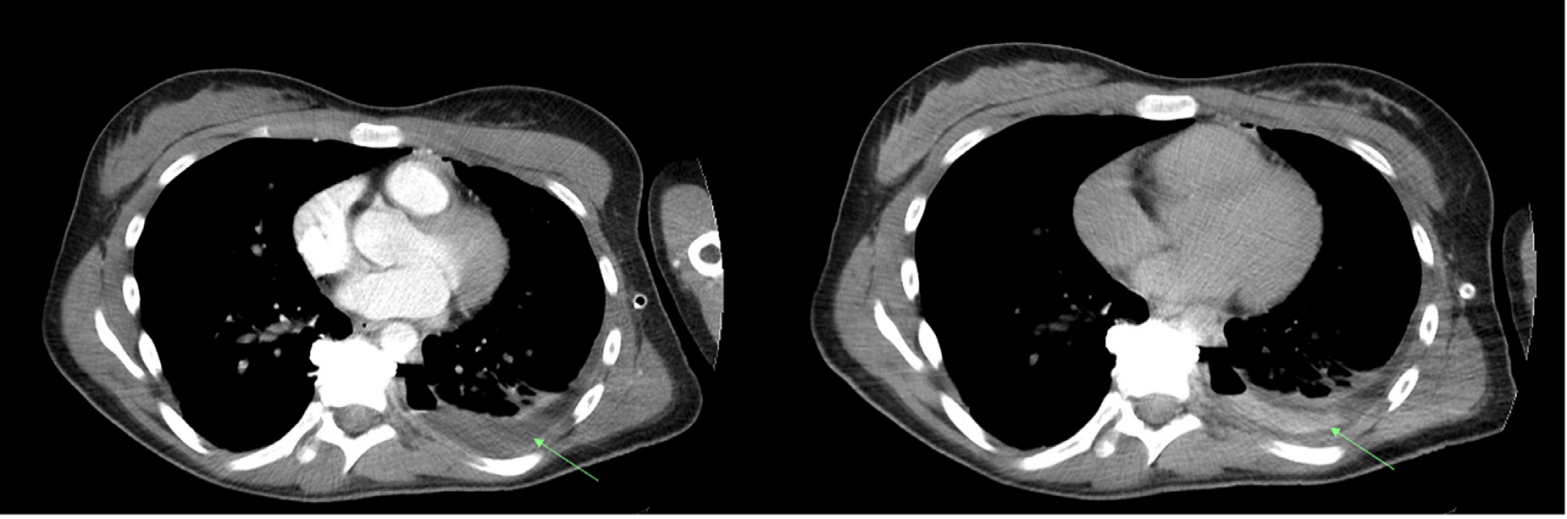
CT chest/abdomen/pelvis without and with contrast, portal venous phase (left) and delayed phase (right). Delayed phase imaging shows contrast opacification within the previously hypodense pleural effusion (arrow), suggestive of extraluminal contrast material entering the pleural space.

**Fig. 3:**
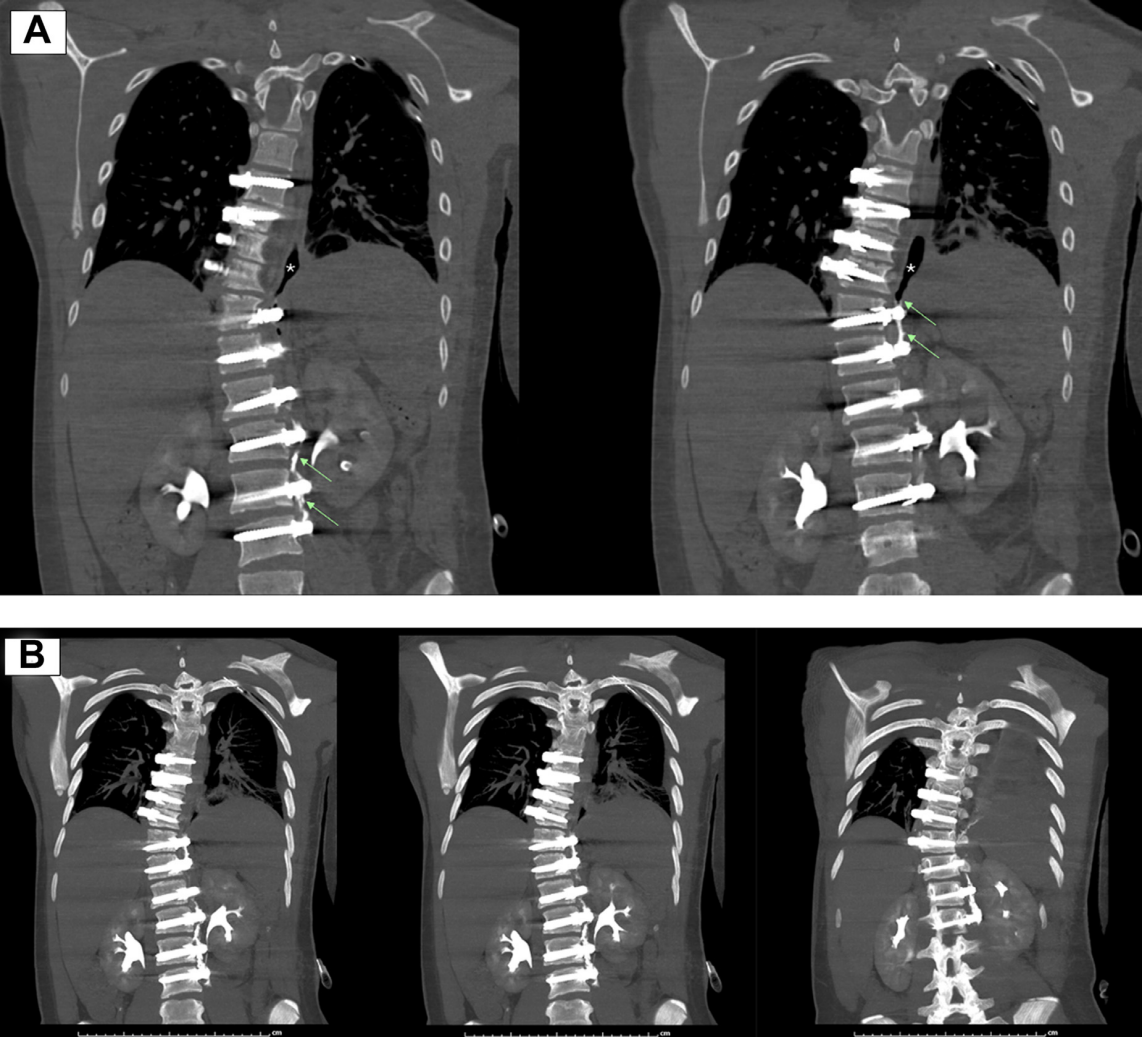
(A) CT chest/abdomen/pelvis without and with contrast, delayed phase imaging demonstrates a tract (arrow) of extraluminal contrast originating from the ureter traveling cranially into the pleural space (*). (B) Maximum intensity projections demonstrating nephropleural fistula tract.

A CT of the abdomen and pelvis was performed with and without contrast which revealed a track of contrast parallel to the left-sided vertebral body tethering screws extending from the level of the mid-left ureter to the left pleural space, without downstream opacification of the distal ureter, compatible with a ureteral-pleural tract/fistula (Fig. 2, Fig. 3). There was no contrast extravasation outside the fistula. Urology was consulted, and a left-sided nephrostomy tube was placed by interventional radiology, which significantly decreased left-sided chest tube output. Follow-up imaging showed near resolution of the left-sided urinothorax. The patient was stabilized and discharged with plans for outpatient ureteral-pleural tract/fistula corrective surgery with urology.

## Discussion

Current literature suggests AVBT complication rate of 18%, with pulmonary etiologies such as pneumothorax and pleural effusion [1] among the most common. In a retrospective review of 140 patients with AIS treated with VTB, Trobisch et al. [3] found an incidence of up to 8% for recurrent pleural effusion postoperatively. Etiology of these pleural effusions is typically hemothorax or chylothorax. Other than this case report, there was only one other reported a case of ureteral injury post-AVBT which resulted in recurrent pleural effusions in a 13-year-old female patient [4].

Nephropleural fistula describes an abnormal connection between the pleural space and the renal collecting system resulting in urine accumulation in the pleural space. This can lead to respiratory distress. Injury to the renal collecting system causes extraluminal extravasation of urine, which tracks through the retroperitoneal space and connects with the pleural space. The mechanism of urine transit has been a cause of debate where one theory suggests the direct transit of urine through a porous diaphragm into the pleural space, whereas another theory advocates for the drainage of urine through the diaphragmatic lymphatic system due to increased retroperitoneal pressure from the adjacent urinoma [5].

The etiology of urinothorax can be divided into 2 broad categories: obstructive uropathy and traumatic injury, with idiopathic injury being the most common [5]. Postsurgical urinothorax is most associated with percutaneous nephrolithotomy [6,7]. However, the case of urinothorax highlighted above documents a rare complication of AVBT, with inadvertent postsurgical connection and fistulous tract between retroperitoneum and the pleural space. Based on clinical history, we noted that the patient developed a mature nephropleural fistula around POD 6, as she developed acute shortness of breath on POD 7, and was found to have a complex left-sided pleural effusion that was later confirmed to be consistent with urinothorax.

The treatment of urinothorax is dependent on its etiology. In the setting of obstructive urinothorax, treatment should be tailored to alleviate the obstruction, as well as thoracentesis to drain the pleural fluid [8]. In our case, the patient responded favorably to an IR-guided percutaneous nephrostomy tube proximal to the nephropleural fistula, which decreased output from her chest-tube. After initial stabilization, she followed up with urology as an outpatient for ureteral-pleural tract/fistula corrective surgery.

As AVBT has only recently become more widely used in treatment of patients with AIS, the full spectrum of associated risks has not been well recorded by literature. Overall, this case highlights ureteral transection and nephropleural fistula as a rare but significant postoperative complication of AVBT. The development of nephropleural fistula and urinothorax are uncommon but necessitates prompt recognition and intervention. Continued analysis of such cases, along with meticulous preoperative planning and intraoperative vigilance, is crucial to improving the safety and efficacy of AVBT procedures.

## Patient consent

Informed consent was obtained from the patient for the publication of this case report, including all associated images and data. All identifying information has been anonymized to maintain confidentiality.

## References

[1] Raitio A, Syvänen J, Helenius I (2022). Vertebral body tethering: indications, surgical technique, and a systematic review of published results. J Clin Med.

[2] Crawford CH, Lenke LG (2010). Growth modulation by means of anterior tethering resulting in progressive correction of juvenile idiopathic scoliosis. J Bone Joint Surg Am.

[3] Trobisch P, Migliorini F, Vanspauwen T, Baroncini A (2022). Pulmonary complications after vertebral body tethering: incidence, treatment, outcomes and risk factor analysis. J Clin Med.

[4] Rathbun JR, Hoernschemeyer DS, Wakefield MR, Malm-Buatsi E, Murray K, Ramachandran V (2019). Ureteral injury following vertebral body tethering for adolescent idiopathic scoliosis. J Pediatr Surg Case Rep.

[5] Toubes ME, Lama A, Ferreiro L, Golpe A, Alvarez-Dobano J, Gonzalez-Barcala F (2017). Urinothorax: a systematic review. J Thorac Disease.

[6] Baugh AD, Youssef E, Hasan SS, Siddiqui NS, Elsamoloty H, Shahrour K (2016). Nephropleural fistula effectively managed with serial thoracentesis: a case report. J Endourol Case Rep.

[7] Hyatt E, Park H, Srinivasa R, Kalva S (2017). Nephropleural fistula. J Vasc Intervent Radiol.

[8] Powers RE, Estrada-Y-Martin RM, Cherian SV (2022). Urinothorax: an under-reported cause of pleural effusions. QJM: Int J Med.

